# New insight into silica deposition in horsetail (*Equisetum arvense*)

**DOI:** 10.1186/1471-2229-11-112

**Published:** 2011-07-29

**Authors:** Chinnoi Law, Christopher Exley

**Affiliations:** 1 The Birchall Centre, Lennard-Jones Laboratories, Keele University, Staffordshire, ST5 5BG, UK

**Keywords:** Biosilicification, biogenic silica, silicic acid, horsetails, callose, PDMPO, fluorescence, acid digestion.

## Abstract

**Background:**

The horsetails (*Equisetum sp*) are known biosilicifiers though the mechanism underlying silica deposition in these plants remains largely unknown. Tissue extracts from horsetails grown hydroponically and also collected from the wild were acid-digested in a microwave oven and their silica 'skeletons' visualised using the fluor, PDMPO, and fluorescence microscopy.

**Results:**

Silica deposits were observed in all plant regions from the rhizome through to the stem, leaf and spores. Numerous structures were silicified including cell walls, cell plates, plasmodesmata, and guard cells and stomata at varying stages of differentiation. All of the major sites of silica deposition in horsetail mimicked sites and structures where the hemicellulose, callose is known to be found and these serendipitous observations of the coincidence of silica and callose raised the possibility that callose might be templating silica deposition in horsetail. Hydroponic culture of horsetail in the absence of silicic acid resulted in normal healthy plants which, following acid digestion, showed no deposition of silica anywhere in their tissues. To test the hypothesis that callose might be templating silica deposition in horsetail commercially available callose was mixed with undersaturated and saturated solutions of silicic acid and the formation of silica was demonstrated by fluorimetry and fluorescence microscopy.

**Conclusions:**

The initiation of silica formation by callose is the first example whereby any biomolecule has been shown to induce, as compared to catalyse, the formation of silica in an undersaturated solution of silicic acid. This novel discovery allowed us to speculate that callose and its associated biochemical machinery could be a missing link in our understanding of biosilicification.

## Background

Silicon is the second most abundant element of the Earth's crust after oxygen and, perhaps surprisingly, its essentiality in biota remains equivocal [1]. The difficulty in ascribing true biochemical essentiality to silicon probably emanates from a lack of demonstration of any silicon-requiring biochemistry and specifically Si-C, Si-O-C, Si-N, et c. bonds in any form of extant life [2]. However, in spite of such limitations the essentiality of silicon in plants remains the subject of rigorous debate [3,4] as do elaborations of the underlying mechanisms. Biosilicification was recently defined as *'the movement of silicic acid from environments in which its concentration does not exceed its solubility (< 2 mM) to intracellular or systemic compartments in which it is accumulated for subsequent deposition as amorphous hydrated silica' *[5] and a number of plants are known biosilicifiers [4]. One of the best known of these are the horsetails, *Equisetum sp.*, and silica deposition in the tissues of these plants has been studied extensively [6-12], perhaps the seminal work in the field being carried out by Perry and Fraser [13]. In this work scanning and transmission electron microscopy was used to illuminate the elaborate and detailed micromorphology and ultrastructure of silicas extracted from different regions of the horsetail, *Equisetum arvense*. The images of silicified stomata and other silica sculptures are truly breathtaking and the level of organisation of silica in the tissues prompted the authors to speculate that *'the silica acts as an in vivo stain, faithfully reproducing the organic matrix skeleton at the microscopic and macroscopic levels without staining'*. Perry and Lu (1992) suggested that the organic matrix in question might be made from polymers of carbohydrates, for example, cellulose [14], and this suggestion was reinforced recently by Fry and colleagues who speculated that the hemicellulose, callose, in horsetail cell walls might be a potential site of silica deposition [15]. Many different biomolecules, often having originally been extracted from biogenic silica, have been shown to accelerate or catalyse silica deposition in saturated solutions of silicic acid [16]. However, biosilicifiers, such as horsetails, harvest silicic acid from solutions which are far from saturation and deposit it as amorphous hydrated silica and it is the elucidation of this mechanism which remains the '*Holy Grail*' of biological silicification research [5].

Herein we have taken inspiration from the work of Perry and Fraser [13] on horsetail and we have used fluorescence microscopy to investigate biosilicification in horsetail and to identify the organic matrix involved in templating silica deposition in this plant.

## Results

### PDMPO as a fluorescent marker of biosilicification

**Figure 1:**
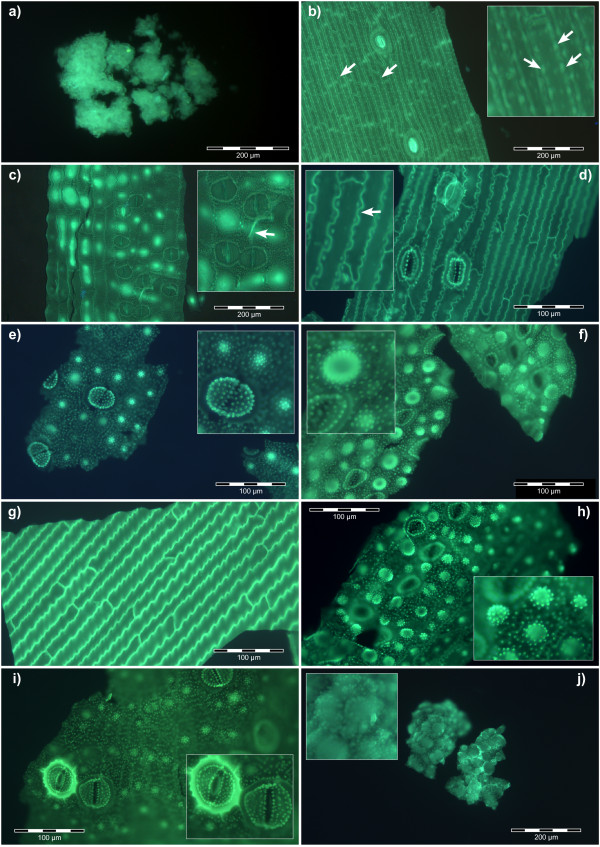
**PDMPO-labelled silica deposition in horsetail**. **a**. Rhizome; **b**. Basal stem, arrows (main and insert) indicate punctate deposits of silica associated with cell walls; **c**. Basal stem, arrow (insert) indicates silica deposition at cell plate between dividing cells; **d**. Basal stem, arrow (insert) indicates punctate deposits of silica associated with highly invaginated cell walls; **e**. Distal stem, showing (main and insert) rosette-like silica structures and heavily silicified stomata; **f**. Node, showing high density of silicified structures including doughnut-like pore (insert); **g**. Node, showing jagged appearance of silica-rich cell walls; **h**. Leaf, showing high densities of rosette-like silica structures; **i**. Leaf, demonstrating the intimate association of silica with stomata (insert); **j**. Spores, showing heavily silicified spores including (insert) punctate deposits of silica on the spore surfaces. Scale bars; 100 μm - d,e,f,g,h,i; 200 μm - a,b,c,j.

Microwave-assisted acid digestion of horsetail, either grown hydroponically in the presence of silicic acid or in plants collected from the wild, resulted in silica deposits and 'skeletons' which were successfully labelled with the fluor PDMPO. Silica was identified in acid digests of all areas of the plant from the rhizome through to spores in the cone. There were no structurally-distinct silica skeletons in the root, only what appeared as diffuse deposits of siliceous materials (Figure 1a). Silica skeletons of basal stem showed epidermal-like cells, 30-40 μm wide and 100-300 μm long, with heavily silicified cell walls and approximately equidistant punctate deposits of silica within the walls which were suggestive of the expected locations of plasmodesmata. Each 'silica cell' included an amorphous, spherical silica deposit between 10 and 20 μm in diameter which had the appearance of a nucleus or vesicle. There were also occasional heavily silicified (as indicated by an enhanced fluorescence) skeletons of stomata, approximately 40 μm wide and 70 μm long, which appeared to be at various stages of differentiation (Figure 1b). In other silica skeletons of basal stem the sections were characterised by many small punctate deposits of silica, <1 μm across, while the stomata, *ca *40-50 μm in diameter, were more numerous, only lightly silicified and many appeared to be linked in pairs. Adjacent epidermal-like cells were *ca *100-200 μm in length and 40-50 μm wide and included highly fluorescent silica deposits which, concomitant with their parent silica cells appeared to be in the process of division (Figure 1c). Some sections of silicified stem showed silica cells which were 100-400 μm in length but without the intracellular, nucleus/vesicle-like deposits seen in other stem sections. The silicified cell walls were heavily invaginated and, again, included punctate and equidistant deposits of silica which as suggested previously may be indicative of the positions of plasmodesmata (Figure 1d). Silica skeletons of distal stem sections were quite different from basal sections in that they were characterised by rosette-like accumulations of silica deposits approximately 20-30 μm in diameter as well as guard cells of stomata studded with silica deposits of *ca *1-2 μm across and resembling 'teeth' where they extended into the stomatal pore (Figure 1e). These silica rosettes appeared to be further elaborated in nodal regions where they formed doughnut-like structures, up to *ca *40 μm in diameter, which gave the distinct impression of being silicified pores (Figure 1f). Other nodal regions showed long, *ca *200-500 μm, epidermal-like cells in which their jagged-in-appearance cell walls were heavily silicified. There were neither punctate silica deposits nor intracellular silica inclusions evident in these structures (Figure 1g). The leaves showed silica skeletons which were very similar to those of the nodal regions though perhaps showing higher densities of the rosette-like silica structures (Figure 1h). Stomata were heavily silicified in some sections of leaf and showed clear anatomical details including an anular ring between the pore-forming guard cells. Again stomata often appeared as pairs connected by silicified threads of varying diameters (Figure 1i). Spores were found to be heavily silicified, being associated with spore walls and present as sub-micron punctate deposits of silica upon individual silicified spores which were between 20 and 40 μm in diameter (Figure 1j). Horsetail grown from rhizomes collected from the wild under hydroponic conditions in the absence of silicic acid grew normally without any obvious requirement for silicon. Acid digestion of tissues from these plants revealed no silica deposits or skeletons.

### PDMPO as a fluorescent indicator of silica formation in vitro

**Figure 2:**
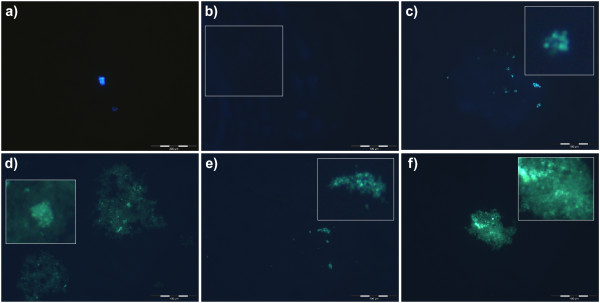
**PDMPO-labelled silica in vitro**. All [PDMPO] are 0.125 μM; All solutions are 20 mM PIPES at pH 7. All [callose] are 5% *w/v*. **a**. PDMPO only; **b**. PDMPO + callose; **c**. PDMPO + callose + 1 mM Si(OH)_4_; the insert shows a close-up of one of the silica clusters; **d**. PDMPO + callose + 2 mM Si(OH)_4_; the insert shows a close-up of the precipitated silica; **e**. PDMPO + 2 mM Si(OH)_4_; the insert shows a close-up of silica; **f**. PDMPO + callose + 4 mM Si(OH)_4_; the insert shows an example of silica formed in this treatment. Scale bars; 100 μm - b-f; 200 μm - a.

Buffer solutions at pH 7 and including 0.125 μM PDMPO showed no green fluorescence indicative of silica and only occasional particles of blue fluorescence probably due to dust or insoluble contaminants in the buffer (Figure 2a). Buffer solutions at pH 7 and including 5% *w/v *callose and PDMPO, but not Si(OH)_4_, showed no green fluorescence while callose was indicated as amorphous blue fluorescence (Figure 2b). Buffer solutions at pH 7 and including 1 mM Si(OH)_4 _(undersaturated) and 5% *w/v *callose showed significant green fluorescence in the presence of PDMPO (Figure 2c). The fluorescent material was primarily made up of aggregates of sub micron-sized particles (Figure 2c insert and arrow) and these appeared to be associated with or occluded within the blue fluorescent callose. Identical solutions in the absence of callose showed no green fluorescence and were similar to image Figure 2a. In buffer solutions at pH 7 which included 2 mM Si(OH)_4 _and 5% *w/v *callose the PDMPO-positive green fluorescence was more extensive than at 1 mM Si(OH)_4 _and included diffuse and particulate materials, the latter again being composed primarily of sub micron-sized particles (Figure 2d). Identical solutions in the absence of callose showed a significantly lesser amount of PDMPO-positive green fluorescence and the fluorescent material was similar in appearance and size to that observed in the presence of callose (Figure 2e). In buffer solutions in which the concentration of Si(OH)_4 _was 4 mM (saturated) there were significant flocs of PDMPO-positive materials and particularly so in those preparations which included 5% *w/v *callose (Figure 2f).

**Figure 3:**
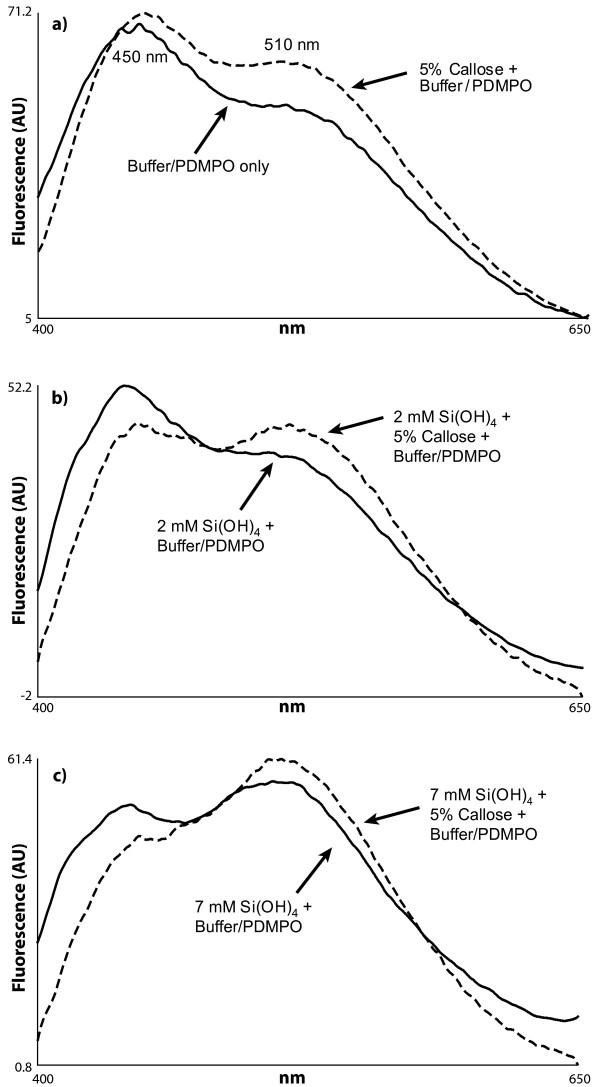
**Emission spectra (Perkin-Elmer LS50B; Ex; 338 nm; Em: 400-650 nm) of 0.125 μM PDMPO in 20 mM PIPES solutions at pH 7 and**; **a**. with or without 5% *w/v *callose; **b**. 2 mM Si(OH)_4 _with or without 5% *w/v *callose; **c**. 7 mM Si(OH)_4 _with or without 5% *w/v *callose.

The presence of silica in an undersaturated (2 mM) solution of Si(OH)_4 _at pH 7 and including 5% *w/v *callose was further supported by fluorescence spectrometry which demonstrated a callose-dependent shift in emission maximum from 450 to 510 nm (Figure 3a,b). That this shift was due to the formation of silica was confirmed in a saturated (7 mM) solution of Si(OH)_4 _under the identical solution conditions (Figure 3c). The silica-dependent shift was significantly more pronounced in the presence than absence of callose.

## Discussion

**Figure 4:**
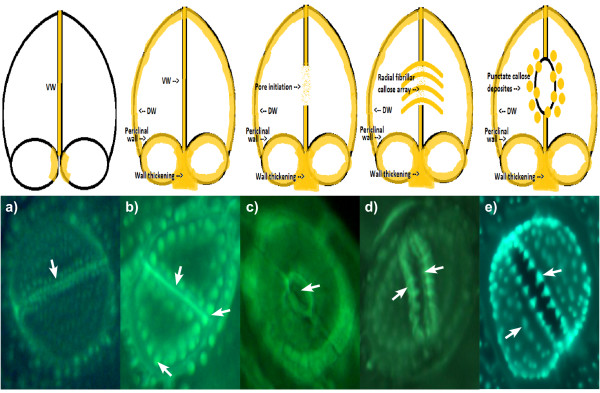
**The deposition of callose (diagrams) and silica (fluorescent images) in the differentiation of stomata in *E. arvense***. **a**. Callose (yellow) and silica (arrow) deposition at the nascent ventral wall (VW) of post-cytokinetic guard cells; **b**. Deposition of callose (yellow) and silica (arrows) in the periclinal wall and dorsal wall (DW) and callose/silica remaining in the ventral wall; **c**. Callose (yellow) and silica (arrow) disappear from the centre of the ventral wall during pore initiation; **d**. Callose (yellow) and silica (arrows) appears as a radial fibrillar array as the stomatal pore is formed; **e**. Upon stomatal pore formation callose (yellow) and silica (arrows) remain as punctate deposits upon the guard cell walls. All stomata are *ca *40 μm in diameter. Information on deposition of callose taken from [19-21].

**Figure 5:**
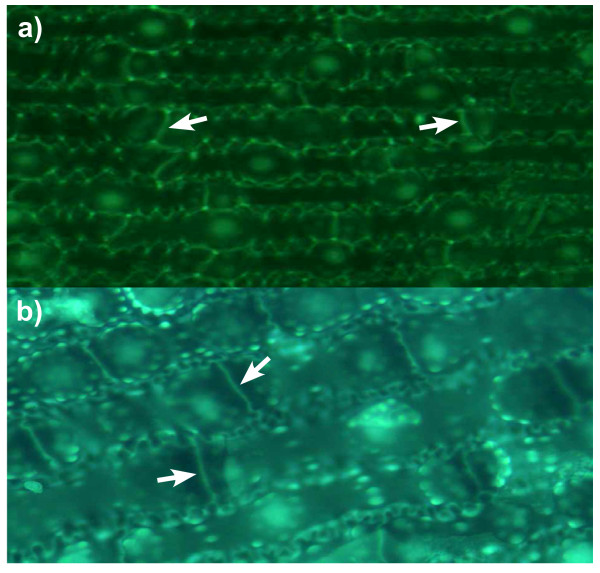
**a,b PDMPO-labelling of silica deposition of cell plates and young cell walls (arrows) forming in cytokinetic cells**.

When fresh or dried samples of horsetail were digested in concentrated acid using a microwave oven all the organic materials associated with the plants were completely dissolved leaving behind elaborate and detailed silica 'skeletons' of the different plant regions. The suspension of these silica remains in buffered solutions at pH 7 which contained the fluorescent probe, PDMPO, enabled their detailed structures to be viewed by fluorescence microscopy (Figure 1). It was of note that horsetail grown hydroponically in the complete absence of added silicic acid grew normally for 10 weeks though without leaving any trace of silica following tissue digestion. While there was no immediate evidence that horsetail required silicon for normal growth it was observed that after 10 weeks of hydroponic culture in the absence of added silicic acid some plants showed wilting and blackening of distal branch tips similar to symptoms of 'silicon-deficiency' observed by Chen and Lewin [17]. However, herein these symptoms appeared simultaneously in parts of the plants where there was evidence of infection by powdery mildew fungus and so it was not clear as to whether they were the result of silicon deficiency or fungal infection [18]. There was no evidence of fungal infection in plants grown in the presence of added silicic acid. While it was clear in horsetail collected locally or grown in silicon-replete hydroponic media that silica was deposited extensively throughout the stem and leaf certain structures showed intense fluorescence which suggested significant silica deposits in these regions. Stomata were often intensley fluorescent (Figure 1) and it was noted that silicification of stomata in horsetail appeared to mirror the known deposition of the hemicellulose, callose, in guard cell differentiation and stomatal pore formation in the related fern, *Asplenium nidus *[19-21]. The observed similarities between the deposition in stomatal structures of callose in *A. nidus *and silica in *E. arvense *were remarkable. For example, in early post cytokinetic guard cells the nascent ventral wall was silicified (Figure 4a). In later examples, the ventral, dorsal and periclinal walls as well as the wall thickenings were are all silicified (Figure 4b). In some stomata silicification was reduced at the centre of the ventral wall as stomatal pore formation was iniated (Figure 4c). Thereafter in further differentiated examples of stomata radial fibrillar arrays of silica were observed on the periclinal wall where stomatal pore formation takes place (Figure 4d). Finally in more mature stomata the wall thickenings were silicified and punctate deposits of silica were observed associated with cell walls (Figure 4e). Annular rings of silica were also observed lining the stomatal pore in more mature stomata (Figure 1i). All of these observations of silica deposition in *E. arvense *have been identified as sites of callose deposition in *A. nidus *(Figure 4) in the recent seminal and detailed studies of Apostolakos and colleagues [19-21]. These very close associations between the known deposition of callose in differentiating stomata and the presence of silica now strongly implicate callose, or possibly, callose in conjunction with an underlying microtubule array, in directing the silicification of stomata in horsetail. Further strong evidence that callose was involved in templating the deposition of silica elsewhere in horsetail was observed in silica skeletons of cells undergoing cytokinesis (Figure 5). Again silica deposition at phragmoplasts and eventually at cell plates and young cell walls dividing daughter cells mirrored the known deposition of callose in cytokinesis [22-24]. In some cells which were at an early stage of division, in some cases before there was any evidence of silica deposition at the phragmoplast, the cytosolic (and perhaps nuclear) fragments of the emerging daughter cells were found to be heavily silicified (Figure 1c). The identity of these silica 'nuclei/vesicles' is a mystery though they may provide evidence for a role for callose in the partitioning of cytosolic and nuclear materials during cell division? The significant deposits of silica within cell walls is supported by the known presence of callose in cell walls of horsetails [12,15,25,26]. In addition, equidistant punctate deposits of silica associated with cell walls may be indicative of, again, the known deposition of callose in plasmodesmata (Figure 1b,d) [27,28]. Finally, the heavily silicified spores (Figure 1j) may also be evidence of the role which is known to be played by callose deposition in plant reproduction [24,29]. Other silica deposits observed in horsetail may also be related to callose deposition. For example, the punctate deposits of silica, sometimes singular and sometimes organised into rosette-like structures, which could be found throughout stem and leaf tissues were identical to those found associated with mature stomata where they are known to mimic callose deposition [30]. In addition the silicified pores of internal diameter 3-5 μm which were identified in leaf tissues (Figure 1f) are not dissimilar to callose lined sieve pores found, for example, in *A. thaliana *[31]. We have successfully applied the fluor PDMPO to demonstrate the deposition of silica in horsetail and in doing so we have identified several novel aspects of biosilicification in horsetail and in particular we have highlighted a potential role for callose in templating silica deposition. Callose biochemistry is, of course, essential in horsetail [15,25], as in many other plants such as the ferns [19-21], and so it is not immediately evident as to how to test whether callose is ultimately required for silica deposition. For example, horsetail is unlikely to grow and/or prosper if the callose synthase gene is knocked out. However, we have been able to support our microscopy evidence linking silica and callose deposition by demonstrating that an undersaturated solution of Si(OH)_4 _(i.e. a solution where the [Si(OH)_4_] ≤ 2 mM) can be induced to form silica in the presence of callose. The formation of silica was confirmed by both fluorescence microscopy (Figure 2) and fluorimetry (Figure 3) and within the usual constraints of such original results we believe that this is the first time that an undersaturated solution of Si(OH)_4 _at room temperature and pressure has been induced to form silica simply by the addition of a biomolecule. When silica extracted from horsetail was added to a 20 mM PIPES-buffered solution at pH 7 which included 0.125 μM PDMPO the emission spectrum changed to give a single emission maximum at *ca *510 nm. This positive control confirmed the known silica-induced shift in the emission spectrum of the fluor PDMPO. A similar shift was also seen for solutions under the same conditions but including 5% *w*/v callose and either 2 or 4 mM Si(OH)_4 _(Figure 3). The former represents an undersaturated solution of Si(OH)_4 _and offered up the first evidence that callose could induce Si(OH)_4 _to autocondense and form silica. However, the *in vitro *evidence was most compelling in preparations containing only 1 mM Si(OH)_4 _when viewed by fluorescence microscopy (Figure 2c). In the absence of callose no silica could be identified by fluorescence microscopy in such preparations while in the presence of callose there were clear and numerous deposits of silica some of which were spherical and approximately 0.5 - 1.0 μm in diameter. Intriguingly the silica bodies were intimately associated with the polymer network of the callose, identified as blue fluorescence, which suggested that the constrained environment generated by the gel-like callose provided the conditions under which an undersaturated solution of Si(OH)_4 _(1 mM) could be 'tricked' into undergoing autocondensation and subsequent growth towards stable aggregates of silica. Callose is a linear homopolymer made up primarily of β-1,3-linked glucose residues which at the concentration used herein, *ca *5% *w/v*, will form a viscoelastic gel [32] within which the orientation of hydroxyl groups on the glucose monomers may be such that they are able to iniate the first steps in the autocondensation of silicic acid as it slowly diffuses within the callose matrix. The hydroxyl groups on the polymer network of callose in some way enable the energy barrier to the autocondensation of Si(OH)_4 _to be overcome and once the first Si-O-Si linkages have been made further condensation reactions can proceed much more easily to eventually build the silica aggregates observed, for example, in Figure 2c. While further experiments will be required to delineate the range of conditions under which callose induces silica formation in undersaturated solutions of Si(OH)_4 _and the exact mechanism by which this is achieved we now have a long sought after biomolecule which can act as a template for silica formation and deposition *in vitro*. If this is also the basis for the mechanism of silica deposition in horsetail then it may also be significant in other callose producing biosilicifiers such as diatoms [33]. If callose is the key then associated biochemistry including enzymes such as callose synthase (potentially catalysing Si-O-Si bond formation) and β-1,3-glucanases (potentially cleaving Si-O-Si bonds) [25] will play a pivotal role in the modelling and remodelling of silica frameworks. The deposition of silica in horsetail has been studied for many decades and we now have a possible mechanism of silica deposition in this plant which could also be a general mechanism of biosilicification.

## Conclusion

The fluor PDMPO has been used to identify silica deposition in horsetail and to provide new insight into silicification in this plant. It was observed that silica deposition in horsetail exactly mirrored the known deposition of callose in the related fern and other plants. Callose was shown to induce the formation and precipitation of silica in undersaturated solutions of silicic acid. This was the first time that this had been demonstrated for any biomolecule and it suggested that callose and perhaps other similar carbohydrates might be key molecules in biological silicification.

## Methods

### Hydroponic culture of horsetail

Horsetail (*Equisetum arvense*) rhizomes were collected locally, washed in ultrapure water (conductivity < 0.067 μS/cm) and subjected to hydroponic culture in 1/6^th ^MS medium in the presence (2 mM) or absence of added silicic acid. The latter media included an additional 8 mM Na^+ ^to account for Si addition as Na_4_SiO_4_. After 10-12 weeks of a 14 h light/10 h dark cycle at 25°C healthy horsetail plants had grown under both sets of conditions.

### Digestion of horsetail materials

Horsetail plants, either collected locally or grown hydroponically, were washed in ultrapure water, allowed to air-dry, cut into discrete 1 cm sections of rhizome/root, basal stem, distal stem, nodal regions and leaves and *ca *0.5 g of each placed in acid-washed 20 mL PFA teflon^© ^vessels. The samples were then digested in a 1:1 mixture of 15.8M HNO_3 _and 18.4M H_2_SO_4 _using a Mars Xpress microwave oven (CEM Microwave Technology Ltd.). The acid digests were clear and, upon dilution with 8 mL of ultrapure water, were filtered and the residues washed several times with further volumes of ultrapure water. Filters were then placed in plastic Petri dishes in an incubator at 37°C to achieve dryness over several days. Dry residues off each filter were then collected into Bijoux tubes and stored in a dry, sealed, perspex cabinet.

### PDMPO labelling of horsetail silica

Silica residues collected from filters were suspended in 20 mM PIPES at pH 7 and containing 0.125 μM 2-(4-pyridyl)-5-((4-(2-dimethylaminoethylaminocarbamoyl) -methoxy)phenyl)oxazole (PDMPO; LysoSensor Yellow/Blue DND-160, 1 mM in DMSO). This intracellular pH probe [34] has been shown to be bound by silica (but not silicic acid) and to emit 'green' fluorescence upon excitation at 338 nm [35-38]. Suspensions were left for 24 h to allow the reaction between silica surfaces and PDMPO to achieve completion after which 50 μL samples were transferred to a cavity slide and viewed using an Olympus BX50 fitted with a BXFLA fluorescent attachment using a U-MWU filter cube (Ex: 333-385 nm; Em: 400-700 nm). A ColourView III digital camera (OSIS FireWire Camera 3.0 digitizer) was used to capture images in conjunction with CELL* Imaging software (Olympus Cell* family, Olympus Soft Imaging solutions GmbH 3.0).

### In vitro preparations of callose and silicic acid

Callose (β-D Glucan, Barley, Sigma, UK) was dissolved at 5% *w/v *in 20 mM PIPES buffer solutions at pH 7 and containing 0, 1, 2, 4 and 7 mM Si(OH)_4 _by warming each preparation in a water bath at 100°C for 60 seconds. Upon cooling to room temperature PDMPO was added to a concentration of 0.125 μM. Equivalent control solutions to which no callose had been added were treated in an identical manner. All solutions were then incubated at room temperature in the dark for 5 days before being examined by fluorescence microscopy, see above, or their emission spectra were determined by fluorimetry (Perkin-Elmer LS50B; Ex; 338 nm; Em: 400-650 nm) as previously described [35].

## Competing interests

The authors declare that they have no competing interests.

## Authors' contributions

CE designed the study and provided training and guidance in experimental methods. CE wrote and prepared the first draft of the manuscript. CL carried out the majority of the experimental work and helped with writing the manuscript.

Both authors have read and approved this manuscript.
